# Genome-wide discovery and functional prediction of salt-responsive lncRNAs in duckweed

**DOI:** 10.1186/s12864-020-6633-x

**Published:** 2020-03-05

**Authors:** Lili Fu, Zehong Ding, Deguan Tan, Bingying Han, Xuepiao Sun, Jiaming Zhang

**Affiliations:** 1 Institute of Tropical Bioscience and Biotechnology, MOA Key Laboratory of Tropical Crops Biology and Genetic Resources, Hainan Bioenergy Center, Chinese Academy of Tropical Agricultural Sciences, Xueyuan Road 4, Haikou, 571101 China; 2 Hainan Academy of Tropical Agricultural Resource, Chinese Academy of Tropical Agricultural Sciences, Xueyuan Road 4, Haikou, 571101 China

**Keywords:** *Spirodela polyrhiza*, lncRNA, Salt treatment, Gene co-expression, ssRNA-Seq

## Abstract

**Background:**

Salt significantly depresses the growth and development of the greater duckweed, *Spirodela polyrhiza*, a model species of floating aquatic plants. Physiological responses of this plant to salt stress have been characterized, however, the roles of long noncoding RNAs (lncRNAs) remain unknown.

**Results:**

In this work, totally 2815 novel lncRNAs were discovered in *S. polyrhiza* by strand-specific RNA sequencing, of which 185 (6.6%) were expressed differentially under salinity condition. Co-expression analysis indicated that the trans-acting lncRNAs regulated their co-expressed genes functioning in amino acid metabolism, cell- and cell wall-related metabolism, hormone metabolism, photosynthesis, RNA transcription, secondary metabolism, and transport. In total, 42 lncRNA-mRNA pairs that might participate in cis-acting regulation were found, and these adjacent genes were involved in cell wall, cell cycle, carbon metabolism, ROS regulation, hormone metabolism, and transcription factor. In addition, the lncRNAs probably functioning as miRNA targets were also investigated. Specifically, TCONS_00033722, TCONS_00044328, and TCONS_00059333 were targeted by a few well-studied salt-responsive miRNAs, supporting the involvement of miRNA and lncRNA interactions in the regulation of salt stress responses. Finally, a representative network of lncRNA-miRNA-mRNA was proposed and discussed to participate in duckweed salt stress via auxin signaling.

**Conclusions:**

This study is the first report on salt-responsive lncRNAs in duckweed, and the findings will provide a solid foundation for in-depth functional characterization of duckweed lncRNAs in response to salt stress.

## Background

Long noncoding RNAs (lncRNAs) are universal in plant and are often regarded as RNA transcripts with length greater than 200 bp but without protein-coding capacity. According to their positions on the genome, lncRNAs are generally classified into the main categories of long noncoding natural antisense transcripts (lncNATs), long intergenic noncoding RNAs (lincRNAs), and long intronic noncoding RNAs [1]. LncRNAs are usually expressed at low levels, thus they are regarded as transcriptional noises for a long time, but emerging evidence has revealed that lncRNAs are important regulatory components responding to various abiotic stresses such as salinity. For examples, over-expression of lncRNA *npc536* enhanced root growth under salinity condition in *Arabidopsis* [2]; another lncRNA *DRIR*, which functioned in water transport and ABA signaling, was characterized as a crucial regulator involved in drought and salt stress [3].

LncRNAs can execute their biological functions in various ways, e.g., they can regulate the expression of genes either in *cis*- or *trans*-acting through sequence complementarity with DNAs or RNAs, epigenetic modification, and promoter activity regulation [1, 4]. In cis-regulation, a flowering time associated lncRNA (*MAS*) positively regulated the transcription of *MAF4* by interacting with *WDR5a*, a key element of COMPASS-like complexes [5]; lncRNA33732, which was activated by *WRKY1* and located 1.8 kb downstream of *RBOH*, prompted the transcription of *RBOH* to increase H_2_O_2_ content in tomato defense responses [6]. In trans-regulation, nitrogen-responsive lncRNA *TAS3* regulated the expression of *NRT2.4* to affect root development under low-nitrogen condition [7]. LncRNAs can also be the cleavage targets of miRNAs [8, 9]. More interestingly, lncRNAs were recently reported to exhibit the novel regulatory mode of target mimics, interacting with miRNAs and influencing associations between miRNAs and their mRNA targets [10]. For example, lncRNA *IPS1*, acting as a target mimic of miR399, can interact with miR399 and depress miR399-regulated cleavage of *PHO2* in *Arabidopsis* for phosphate (Pi) uptake [11].

With the high-speed development of next-generation sequencing, hundreds of lncRNAs have been associated with salt stress in plants through transcriptome re-assembly. In cotton (*Gossypium hirsutum*), a total of 1117 unique lncRNAs were identified, and 44 were differentially expressed under salt treatment [12]. In maize (*Zea mays*), a total of 48,345 distinct lncRNAs were identified, of which 1710 were responsive to both salt and boron stress [13]. In *Medicago truncatula*, a total of 7874 and 7361 lncRNAs were identified from salt-treated root and leaf samples, respectively [14]. In wheat (*Triticum aestivum*), 44,698 lncRNAs were detected throughout the genome by analysis of 52 RNA-seq datasets, and ~ 37% of them were affected by salt [15]. These findings suggest that lncRNAs play an important role in plants under salt condition. However, to date, no comprehensive surveys of salt-responsive lncRNAs have been reported in duckweed.

Duckweeds are a family of small floating aquatic plants that grow fast and have high starch contents and nutrient-uptake rates; therefore, they have attracted broad interest in the application of livestock feed, bio-ethanol production, and wastewater treatment [16, 17]. However, duckweeds are highly sensitive to salt, which greatly restricts the growth and development. Under salt condition, the activities of photosystem I (PSI) and PSII, together with the overall activity of electron transport chain, were dramatically decreased, while the production of reactive oxygen species (ROS) was greatly increased in *Lemna gibba* [18]. In addition to photosynthetic pigment, salt treatment significantly inhibited plant growth but greatly enhanced hydrogen peroxide (H_2_O_2_) and malondialdehyde (MDA) contents in *Spirodela polyrhiza* [19, 20]. Accordingly, ROS scavenging system was triggered to protect against oxidative damage, because many anti-oxidative enzymes including catalase (CAT), superoxide dismutase (SOD), ascorbate peroxidase (APX), and peroxidase (POD) were greatly induced [19, 20]. Salt stress also dramatically decreased the capacity of nitrogen and phosphorus removal. Moreover, this influence was strength-dependent since higher concentrations and longer periods of salt stress caused greater inhibition of nitrogen and phosphorus removal and more injury to duckweeds [21]. These findings provide useful insights into the responses of duckweeds to salt stress. However, these previous studies have primarily focused on the influences of salt stress at the physiological level, while the roles of lncRNAs in the salt stress response of duckweeds remain largely unknown.

In this work, strand-specific RNA sequencing (ssRNA-seq) was employed to examine the transcriptomic changes of duckweed *S. polyrhiza* in response to salt stress. Afterwards, salt-responsive lncRNAs were systematically discovered, their basic characterizations and expression trends were examined, and their putative functions were studied. These results will expand our knowledge of lncRNAs in duckweeds under salt condition, and lay a solid foundation for in-depth functional characterization of these lncRNAs.

## Results

### Salt response and ssRNA-seq of duckweed

**Fig. 1:**
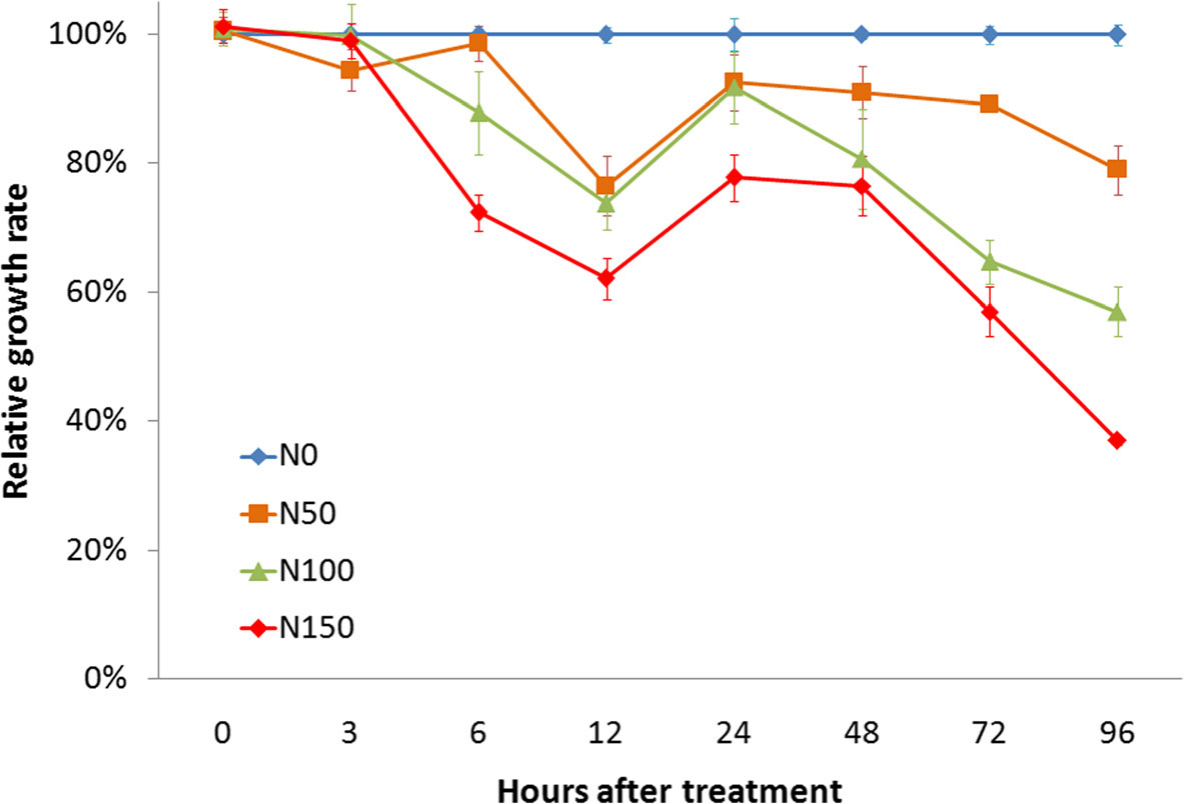
Inhibition of relative growth rate upon salt stress in duckweed. N0 (control), N50, N100, and N150 represent the salt concentrations of 0, 50, 100, and 150 mM NaCl, respectively. The relative growth rates under salt treatment were calculated relative to that under control, which was normalized as 1 at each time-point along with the salt stress

The influence of salt treatment on duckweed growth was investigated under three different NaCl concentrations for a period of 96 h (Fig. 1). Compared with the control (N0), the relative growth rate (RGR) under 50 mM NaCl (N50) treatment gradually decreased from 0 h to 12 h, then recovered a little at 24 h and decreased again until 96 h. Although similar trends were observed under 100 mM (N100) and 150 mM NaCl (N150) treatments, the RGR values dropped more steeply than that of N50 (Fig. 1). These results suggested that duckweed growth was greatly inhibited by salt stress, and this effect became more serious upon increase of salt concentration and extension of treatment period.

To explore the lncRNAs involved in salt stress, duckweed samples were collected at 0, 6, 12, and 24 h, respectively, under 100 mM NaCl treatment and then subjected to ssRNA-seq sequencing. After removing low-quality reads and sequence adapters, a total of ~ 860 million clean reads were generated, of which ~ 83.7% were aligned to the duckweed reference genome.

### Identification and characterization of lncRNA in duckweed

In total, 61,428 transcripts with 1771 bp on average were generated by transcriptome reconstruction of all RNA-seq data. Of which, 54,635 transcripts overlapping with 19,623 protein-encoding genes (representing all of the annotated duckweed genes) were removed. The 6793 remaining transcripts were preliminarily screened by a basic filtering with five steps (e.g., minimal reads coverage ≥3, transcript length ≥ 200 bp, Fig. S1), and further filtered by their coding potential and only the transcripts without protein-encoding capacity were retained. Ultimately, 2815 reliably expressed novel lncRNAs, comprising 566 anti-sense lncRNAs and 2249 intergenic lncRNAs, were discovered based on their positions on the duckweed genome.

**Fig. 2:**
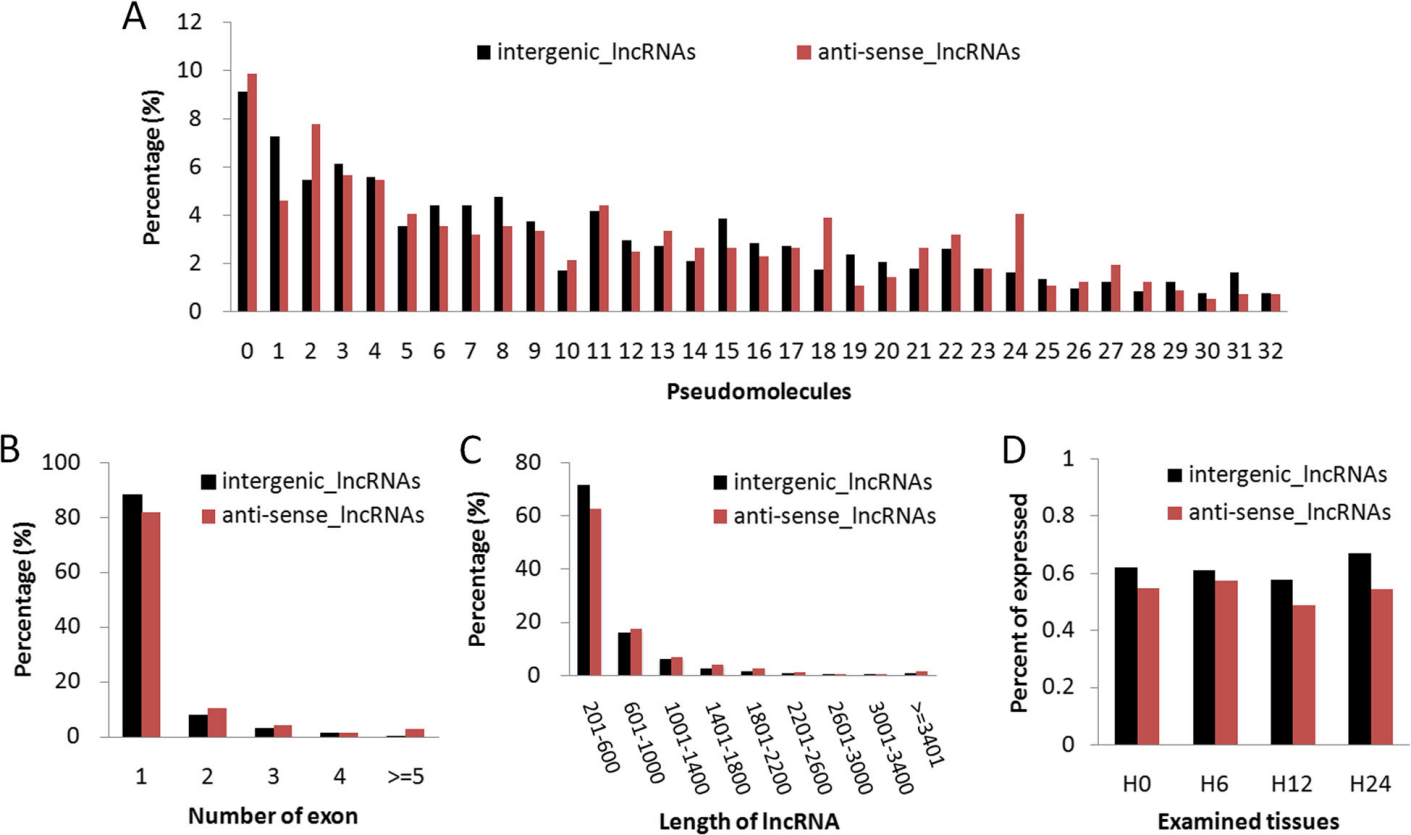
Features of duckweed lncRNAs under salt stress. Percentages of intergenic lncRNAs and anti-sense lncRNAs in pseudo-molecule location (**a**), exon number (**b**), transcript length (**c**), and expression level (**d**), respectively

Characteristics of duckweed lncRNAs, including their distribution on pseudo-molecules (chromosomes), exon number, transcript length, and expression level, were investigated for anti-sense and intergenic lncRNAs, respectively (Fig. 2). Although these two types of lncRNAs were dispersed evenly in most pseudo-molecules, a few exceptions were observed: e.g., higher proportions of anti-sense lncRNAs were found in pseudo-molecule 2, 18, and 24, while higher percentages of intergenic lncRNAs were observed in pseudo-molecule 1, 8, 15, and 31 (Fig. 2a). Similar distribution trends were observed regarding exon number and transcript length: the majority (~ 84%) of intergenic and anti-sense lncRNAs contained only one exon, approximately 10, 3, and 1% contained two, three, and four exons, respectively, and the remaining (~ 2%) contained no less than five exons (Fig. 2b); about two-thirds of lncRNAs ranged between 201 and 600 nucleotides (nt) with median lengths of 406 and 462 nt for intergenic and anti-sense lncRNAs, respectively (Fig. 2c). Overall, the percentage of expressed intergenic lncRNAs (FPKM > 1) was greater than that of anti-sense lncRNAs in all samples (Fig. 2d), suggesting that intergenic lncRNAs were more likely to be expressed than anti-sense lncRNAs. Taken together, these findings provide a general survey of the features of duckweed lncRNAs under salt stress.

### Determination of differentially expressed (DE) lncRNAs

**Fig. 3:**
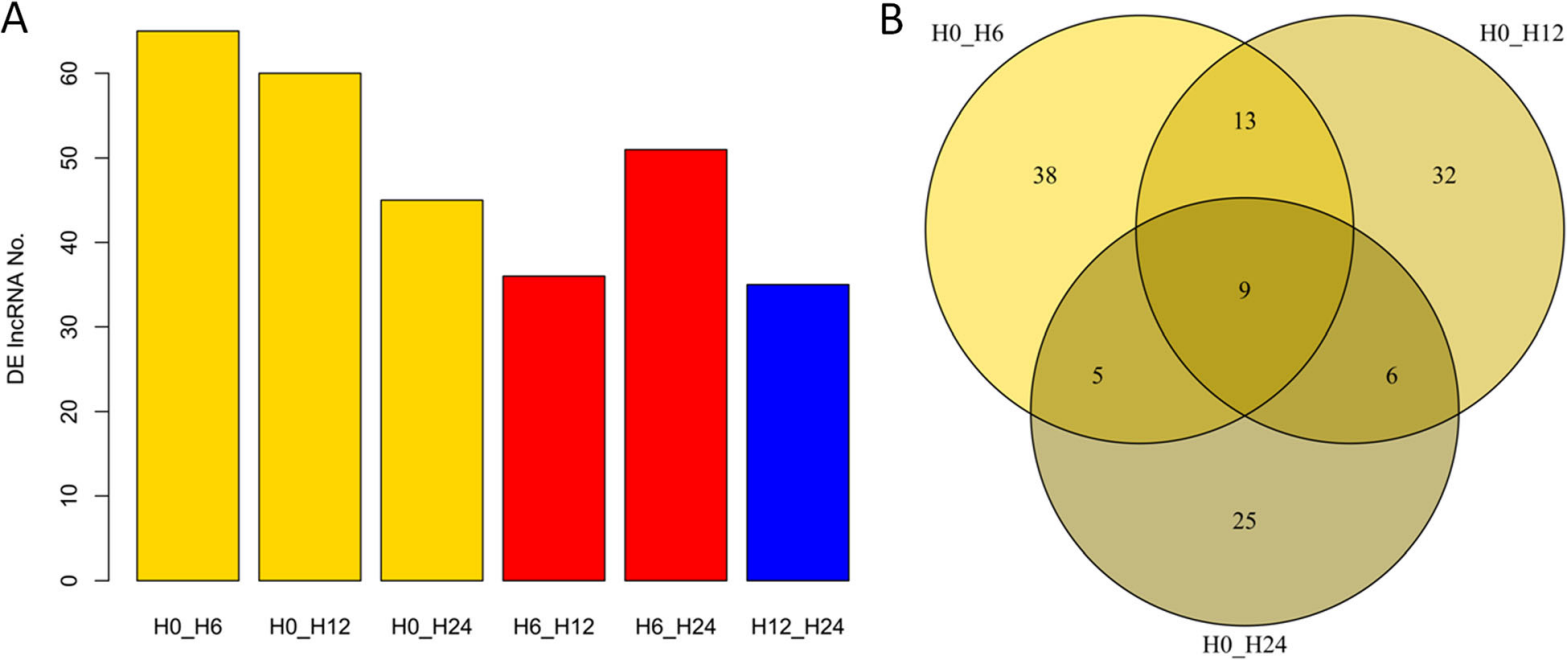
Transcriptomic profiling of duckweed lncRNAs upon salt treatment. DE lncRNAs detected by pair-wise comparison among four samples (**a**) and their Venn diagrams (**b**). H0, H6, H12, and H24 represent the samples collected at 0, 6, 12, and 24 h upon salt stress, respectively

To reveal the transcriptional response of lncRNAs to salt stress, DE lncRNAs were identified pair-wisely among samples. It seems that lncRNAs were prone to be differentially expressed at the early stages of salt treatment, since the number of DE lncRNAs became gradually lower at 6 h (65), 12 h (60), and 24 h (45) when compared with 0 h (Fig. 3a). However, when compared with 6 h, the number of DE lncRNAs observed at 24 h (51) was about 1.5-fold than that at 12 h (36).

In total, 185 DE lncRNAs were found under salt treatment. Of which, 38, 32, and 25 were exclusively identified in comparisons of H0_H6 (0 h vs 6 h), H0_H12 (0 h vs 12 h), and H0_H24 (0 h vs 24 h), and only nine were commonly found in these three comparisons (Fig. 3b). These findings suggest that lncRNAs associated with salt treatment function in a temporal-specific pattern.

### Functional prediction of DE lncRNAs in trans-regulation

**Fig. 4:**
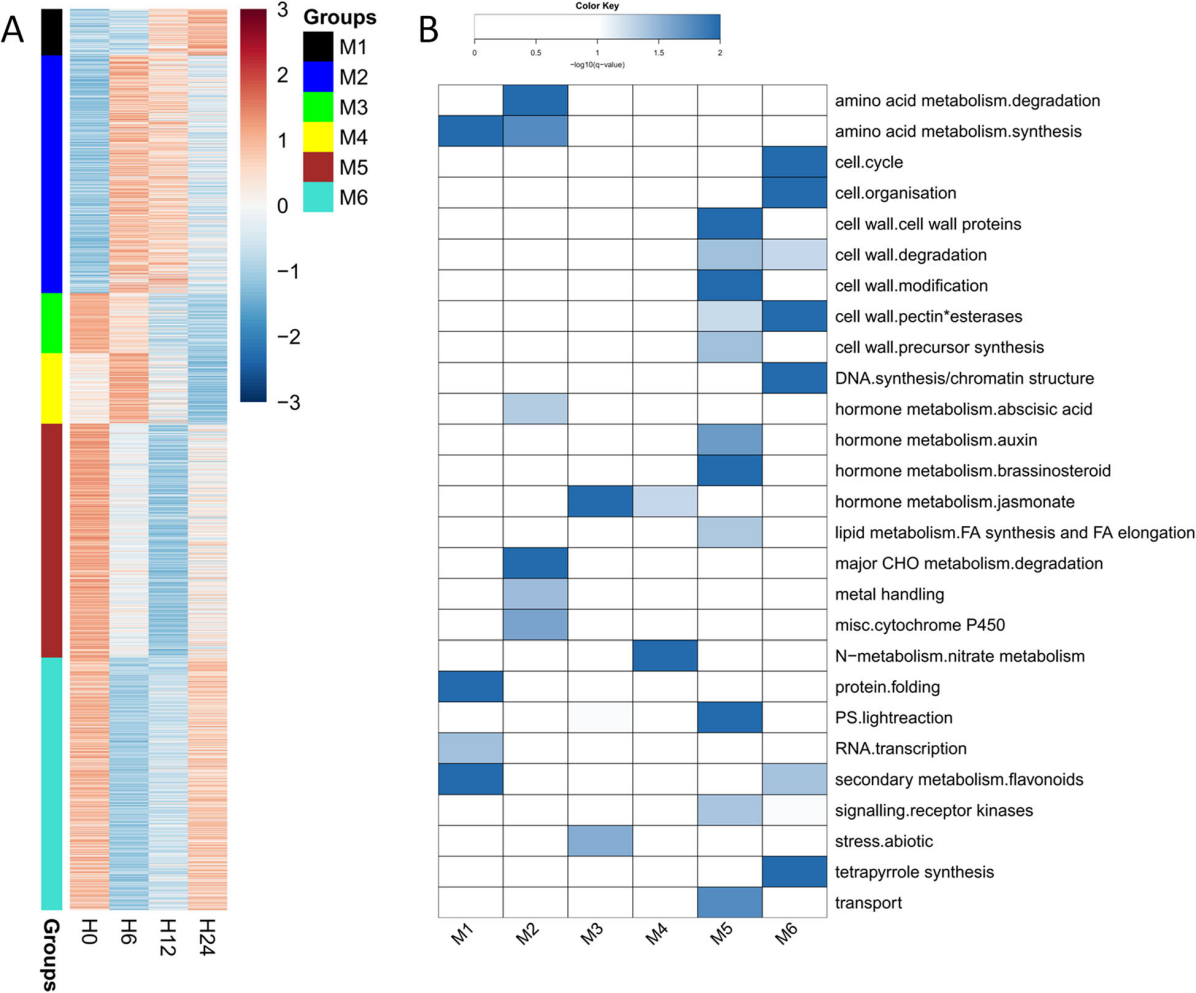
Analysis of lncRNAs and their co-expressed mRNAs. **a** Expression profile of DE lncRNAs and mRNAs that were mainly assigned into six groups (M1-M6). **b** Functional enrichment analysis of each group shown in (**a**)

To characterize the potential function of salt-responsive lncRNAs in trans-regulation, all 185 DE lncRNAs and 2156 DE genes were chosen for co-expression analysis. A total of six co-expressed groups (M1-M6) were found according to their expression trends (Fig. 4a). Group M1 contained 24 lncRNAs, and the lncRNAs/genes in this group were gradually induced from 0 h to 24 h upon salt stress. Functional enrichment analysis showed that these lncRNAs/genes were significantly enriched in amino acid metabolism, protein folding, RNA transcription, and secondary metabolism (Fig. 4b).

There were 50 lncRNAs in group M2. These lncRNAs and their co-expressed genes were rapidly induced at 6 h and 12 h but then declined at 24 h after salt stress. They were enriched in amino acid metabolism, major CHO metabolism, abscisic acid (ABA), and cytochrome P450 (Fig. 4b).

There were 14 and 13 lncRNAs in groups M3 and M4, respectively. Although the expression of lncRNAs/genes in these two groups was commonly decreased at 12 h and 24 h, the lncRNAs/genes in group M3 showed highest expression levels at 0 h whereas those in group M4 were expressed highest at 6 h (Fig. 4a). The enriched categories included abiotic stress in M3, nitrate metabolism in M4, and jasmonate of hormone metabolism in both M3 and M4 (Fig. 4b).

Groups M5 and M6 contained 31 and 36 lncRNAs, respectively. Although similar expression patterns were observed in these two groups (e.g., expressed highest at 0 h and then decreased at 6 h and 12 h), the expression levels were much lower in M5 than in M6 at 24 h (Fig. 4a). The enriched categories of M5 included cell wall related metabolisms, hormone metabolisms (such as auxin and brassinosteroid), FA synthesis and FA elongation, photosynthesis, receptor kinases signaling, and transport; while those of M6 included cell cycle and cell organization, cell wall degradation, DNA synthesis and chromatin structure, secondary metabolism, and tetrapyrrole synthesis (Fig. 4b).

Taken together, these findings suggested that the DE lncRNAs involved in trans-acting regulation mainly participated in amino acid metabolism, cell- and cell wall-related metabolism, hormone metabolism, photosynthesis, RNA transcription, secondary metabolism, and transport under salt treatment.

### Functional prediction of DE lncRNAs in cis-regulation

To characterize the function of salt-responsive lncRNAs in cis-regulation, their adjacent protein-encoding genes, which were placed 10 kb upstream and 100 kb downstream of lncRNAs, were chosen to conduct co-expression analysis. The highly co-expressed and closely located lncRNA-mRNA pairs were in cis-regulation relationships and deserved to be further studied.

**Fig. 5:**
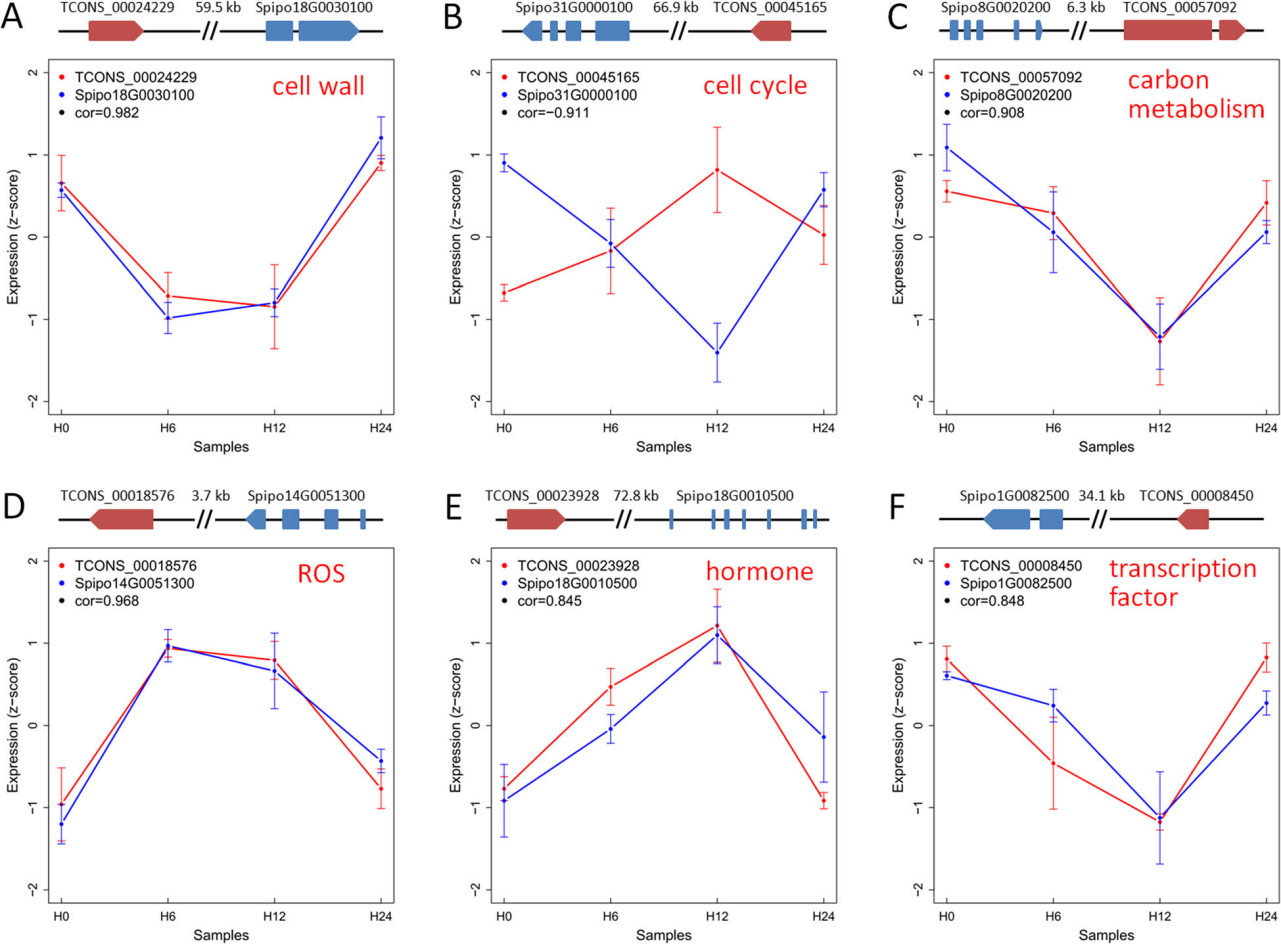
Expression coordinance of lncRNAs and their adjacent genes in cis-regulation. Structure and expression pattern of six lncRNA-mRNA pairs involved in (**a**) cell wall, (**b**) cell cycle, (**c**) carbon metabolism, (**d**) ROS, (**e**) hormone, and (**f**) transcription factor. Expression levels were normalized as Z-scores and presented as means ± standard deviation (*n* = 3)

In total, 42 lncRNA-mRNA pairs probably associated with cis-acting regulation were found (Table S2). Of which, TCONS_00036371 was located 49,867 bp upstream of Spipo24G0014100 encoding a cellulose synthase, TCONS_00024229 was located 59,575 bp upstream of Spipo18G0030100 participating in lignin biosynthesis (Fig. 5a), and TCONS_00045165 was located 66,903 bp upstream of Spipo31G0000100 encoding a cyclin D-type protein involved in cell proliferation (Fig. 5b). Those data implied that these lncRNAs played a major role in cell wall and cell cycle in response to salt treatment.

TCONS_00029753 was spaced 23,667 bp upstream of Spipo2G0088600 participating in starch degradation, and TCONS_00057092 was spaced 6365 bp downstream of Spipo8G0020200 related to photosynthesis (Fig. 5c), suggesting that these two lncRNAs were involved in carbon metabolism under salt stress.

TCONS_00018576 was placed 3725 bp downstream of Spipo14G0051300 encoding a chloroplastic lipocalin against reactive oxygen species (ROS, Fig. 5d), and TCONS_00037548 was spaced 87,103 bp upstream of Spipo26G0017400 participating in redox homeostasis, indicating that these two lncRNAs were involved in ROS metabolism upon salt treatment.

In addition, several lncRNAs associated with hormone metabolism and transcription factors were found. For examples, TCONS_00023928 was located 72,842 bp upstream of Spipo18G0010500 involved in ABA signaling (Fig. 5e), and TCONS_00042227 was spaced 49,912 bp upstream of Spipo3G0088700 involved in auxin response; while TCONS_00045028 was located 10,730 bp upstream of Spipo31G0005500 encoding an ARF transcription factor, TCONS_00008450 was spaced 34,104 bp upstream of Spipo1G0082500 encoding a C2C2(Zn) transcription factor (Fig. 5f), and TCONS_00060414 was placed 85,167 bp upstream of Spipo9G0002000 encoding a HSF transcription factor.

Together, these findings strongly indicated that the cis-acting DE lncRNAs might play major roles in regulation of their adjacent genes related to cell wall, cell cycle, carbon metabolism, ROS regulation, hormone metabolism, and transcription factors in response to salt stress.

### Functional prediction of DE lncRNAs acting as miRNA targets

LncRNAs can act as competitive targets of miRNAs to influence their regulatory efficiency. Therefore, it is of great interest to survey the possibility of salt-responsive lncRNAs functioning as targets of miRNAs (especially those with well-known functions).

**Fig. 6:**
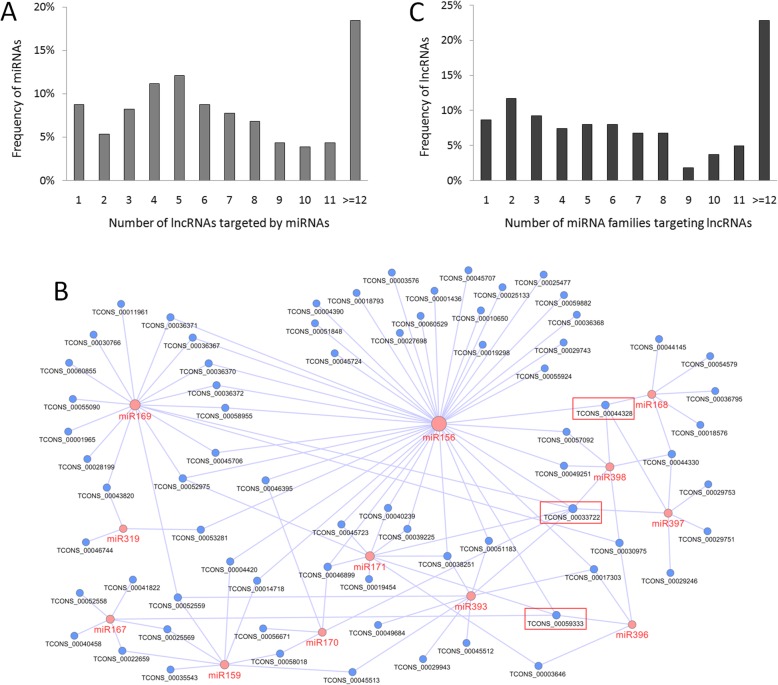
Investigation of lncRNAs acting as miRNA targets. **a** Frequency of miRNAs targeting on lncRNAs. **b** Interactive networks of miRNA and lncRNAs, which were represented by pink and blue cycles, respectively. Node sizes were determined based on the degrees/connections to others. Three lncRNAs with most connections to miRNAs were marked by red box. **c** Frequency of lncRNAs targeted by miRNA families

A total of 162 DE lncRNAs were predicted to be targeted by 388 miRNAs derived from 206 families (Table S3). The number of lncRNAs targeted by miRNAs varied from one to forty, and the high-frequency miRNAs usually targeted four to five lncRNAs (Fig. 6a). Notably, miR156, which is well-known to participate in many abiotic stresses including salt, cold, and drought [22], targeted as many as 40 lncRNAs, strongly indicating that this miRNA might play an important role in duckweed salt stress via miRNA-lncRNA interaction. In addition to miR156, several other salt-responsive miRNAs, including miR169, miR171, and miR393 [22], were found. The lncRNAs targeted by these miRNAs might be useful candidates functioning in salt stress, and their miRNA-lncRNA interaction networks were presented in Fig. 6b.

The number of miRNA families possessing the ability to target lncRNAs was also investigated (Fig. 6c). The proportion of lncRNAs targeted by one to eight miRNA families was similar, but far fewer lncRNAs were targeted by nine or more miRNA families. Notably, TCONS_00033722 was probabaly targeted by as many as 68 miRNAs including a few salt-responsive miRNAs, e.g., miR156, miR169, miR171, and miR393 (Fig. 6b). TCONS_00044328 and TCONS_00059333 might be also targeted by miR156 as well as other salt-responsive miRNAs such as miR167, miR168, and miR171 (Fig. 6b). Taken together, these results strongly indicated the involvement of these three lncRNAs in duckweed salt stress with the participation of miRNA regulation.

### Expression confirmation of lncRNAs and genes

**Fig. 7:**
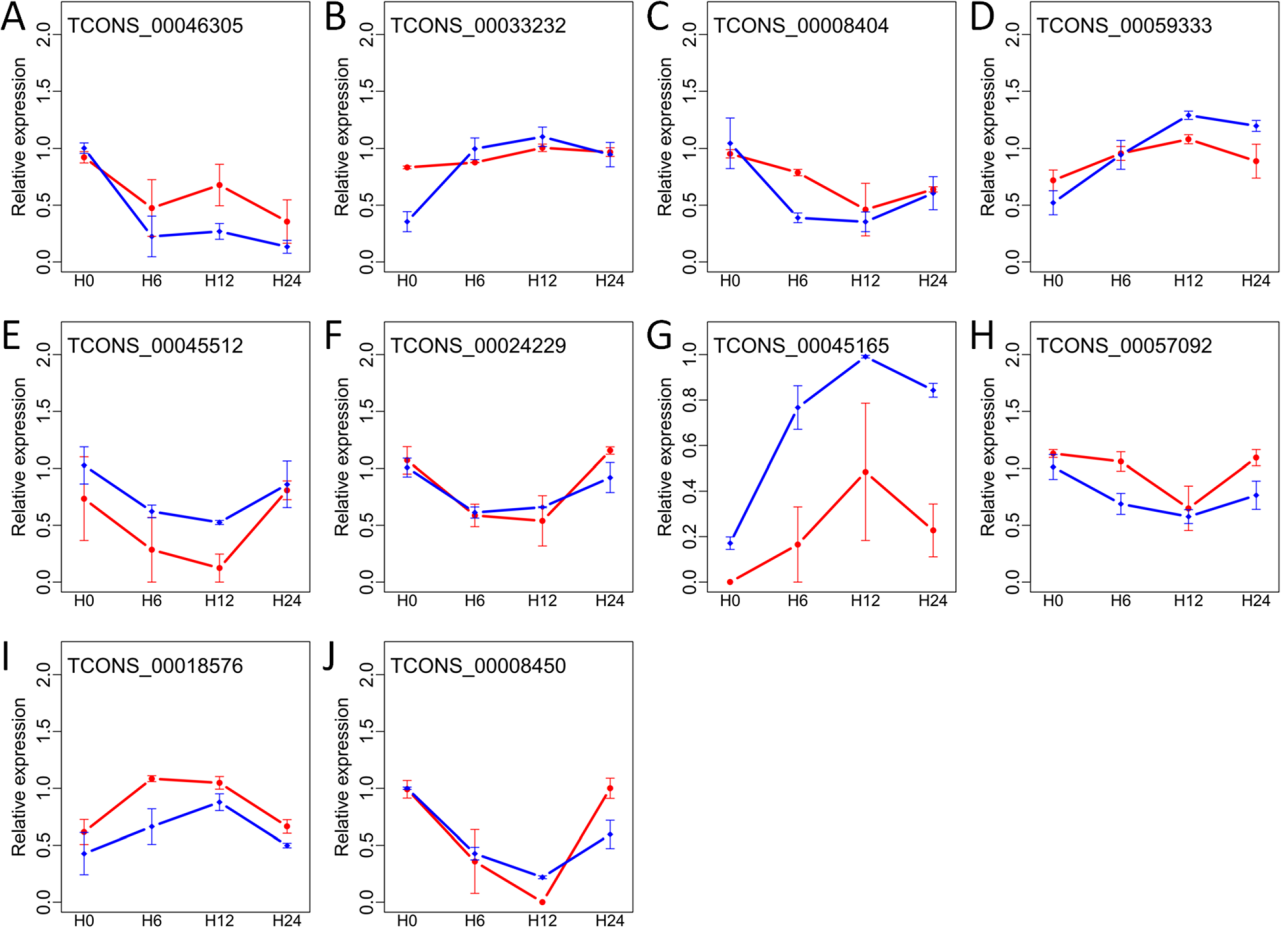
Expression validation of ten lncRNAs by qRT-PCR. These lncRNAs were taken part in trans-acting regulation (**a-c**), miRNA target (**d-e**), and cis-acting regulation (**f-j**), respectively. Expression levels were normalized by the maximum value among samples and shown as mean ± standard deviation (*n* = 3) for qRT-PCR (blue lines) and ssRNA-seq (red lines), respectively

In total, ten key lncRNAs participated in trans-regulation, cis-regulation, or as miRNA targets, and five genes co-expressed with lncRNAs were examined by qRT-PCR. The correlation coefficient varied from 0.75 to 0.97 between qRT-PCR and ssRNA-seq methods (Fig. 7 and Table S1), suggesting the reliable expression of lncRNAs and genes detected by ssRNA-seq data.

## Discussion

### LncRNA is a crucial player in duckweed salt stress

LncRNA has been illustrated to play an important regulatory role in salt stress response of many species, including cotton [12], maize [13], *M. truncatula* [14], and wheat [15]. However, the roles of lncRNAs have so far not been reported in duckweed upon salt treatment, which greatly inhibited the vegetative growth of duckweed [23]. In the present work, a total of 2815 lncRNAs (including 566 anti-sense lncRNAs and 2249 intergenic lncRNAs) were systematically identified in duckweed under salinity condition using ssRNA-seq technology. The number of lncRNAs was about 2.5-fold higher than that reported in cotton [12], but much less than that identified in *M. truncatula*, maize, and wheat [13–15]. These results, to a certain extent, reflected the influences of sequencing depth, plant species, and applied parameters on lncRNAs discovery.

The general features of duckweed lncRNAs were subsequently revealed. Most lncRNAs were shorter than 1000 nt and only contained 1–2 exons (Fig. 2), similar to those reported in *M. truncatula* and maize [13, 14]. Overall, anti-sense and intergenic lncRNAs were similarly distributed on duckweed pseudo-molecules. However, the percentages of expressed anti-sense lncRNAs were lower than those of intergenic lncRNAs in all samples (Fig. 2d), which is inconsistent with those reported in cassava [24].

LncRNAs were reported to function in a tissue-specific or temporal-dependent manner [15, 24, 25]. In our work, a total of 185 lncRNAs were differentially expressed in response to salt, of which 38, 32, and 25 were exclusively identified at 6, 12, and 24 h of salt stress compared with the control (Fig. 3b), indicating that the expression of salt-responsive lncRNAs was rigorously regulated in a temporal-dependent manner, in accordance with those described previously [25]. Together, these results highly suggest that lncRNA is a crucial player in duckweed in response to salt stress.

### Functional analysis of duckweed lncRNAs upon salt treatment

LncRNAs can execute their functions in *cis*-acting to regulate the expression of their adjacent genes. Notably, in tomato, lncRNA33732 activated the expression of *RBOH* located ~ 1.8 kb upstream of lncRNA33732 to increase H_2_O_2_ content in early defense responses [6]; similarly, maize lncRNA *Vgt1* depressed the expression of *ZmRap2* located ~ 70 kb downstream of *Vgt1* and was involved in flowering time [26]. Here, a set of 42 lncRNA-mRNA pairs participating in *cis*-acting regulation were found, and a few of them were further confirmed by qRT-PCR (Table S1). The neighboring genes regulated by lncRNAs were related to cell wall, cell cycle, carbon metabolism, and ROS regulation. For examples, TCONS_00024229 was located 59.6 kb upstream of Spipo18G0030100 participating in lignin biosynthesis (Fig. 5a), TCONS_00057092 was placed ~ 6.4 kb downstream of Spipo8G0020200 associated with photosynthesis (Fig. 5), cand TCONS_00018576 was spaced ~ 3.7 kb downstream of Spipo14G0051300 coding a chloroplastic lipocalin against ROS (Fig. 5d), in accordance with the previous studies that genes referred to cell wall, photosynthesis, and ROS signaling were greatly affected by salt [27, 28]. Besides, a few neighboring genes related to hormone metabolism and transcription factors were also found in lncRNA-mRNA pairs associated with *cis*-acting regulation. Specifically, TCONS_00023928 was located 72.8 kb upstream of Spipo18G0010500 participating in ABA signaling (Fig. 5e), and TCONS_00045028 was located 10.7 kb upstream of Spipo31G0005500 encoding an ARF transcription factor, supporting the involvement of hormone and transcription factor genes in salt stress [29, 30].

LncRNAs can also execute their roles in *trans*-acting to affect the expression of genes located far away [7, 31]. Thus, in the present work, co-expression network analysis was employed to investigate the roles of lncRNAs based on the enriched functions of their co-expressed genes. Totally 31 lncRNAs, together with 564 co-expressed genes, were clustered in group M5, and their expression levels were significantly declined from 6 h to 24 h, in accordance with the growth inhibition of duckweed upon salt stress (Fig. 1 and Fig. 4a). Enrichment analysis revealed that these salt-responsive lncRNAs were mainly involved in cell wall, lipid metabolism, and photosynthesis, which might be one of the explanations for the inhibited growth of duckweed under salt [27, 32]. Salt-responsive lncRNAs were also co-expressed with genes related to hormone metabolism, major CHO metabolism, RNA transcription, abiotic stress, and transport (Fig. 4b), indicating similar functions of lncRNAs in duckweed under salinity condition. Interestingly, comparable roles of lncRNAs were observed in other plant species: in wheat, Shumayla et al. [15] showed that lncRNAs were co-expressed with various transcription factors and several ABA biosynthesis genes under salt; in cotton, Deng et al. [12] concluded that lncRNAs might regulate protein-coding genes related to carbohydrate metabolism, transcription, cellular component, stress response, and transport in response to salt stress; in *M. truncatula*, Wang et al. [14] reported that lncRNAs were involved in cellular component, carbohydrate metabolism, signal transduction, and transcription upon salt treatment. Besides, salt-responsive lncRNAs were also co-expressed with genes referred to amino acid metabolism, cell cycle, and secondary metabolism (Fig. 4b). Although no such functions of salt-responsive lncRNAs have been reported, several studies have confirmed that genes related to amino acid metabolism, cell cycle, and biosynthesis of secondary metabolites were greatly altered under salinity condition [33, 34], providing us a high possibility to further characterize lncRNAs with similar functions in duckweed in the future.

### lncRNA-miRNA-mRNA networks involved in salt stress via auxin signaling

**Fig. 8:**
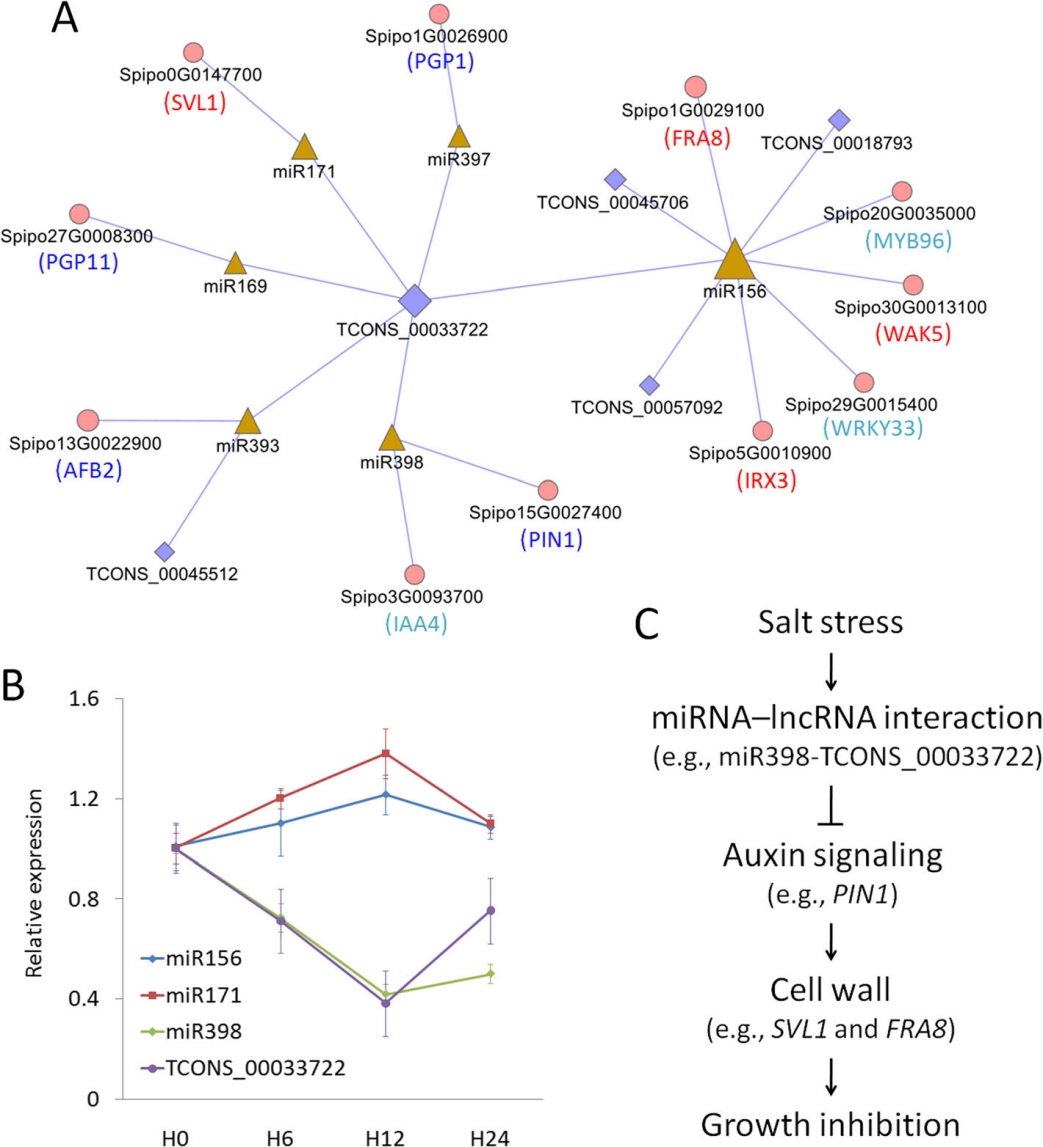
Network analysis of lncRNAs, miRNAs, and mRNAs. **a** An interactive network of lncRNAs, miRNAs, and mRNAs referred to duckweed salt stress via auxin signaling. LncRNAs, mRNAs, and miRNAs were indicated by blue diamonds, pink cycles, and yellow triangles, respectively. Node sizes were determined based on the degrees/connections to others. **b** Coordinated expression of TCONS_00033722 and three miRNAs (miR156, miR171, and miR398). Data were shown as mean ± standard deviation (*n* = 3) and the expression of H0 was normalized as 1. **c** A schematic model of lncRNA-miRNA-mRNA interaction for growth inhibition of duckweed upon salt stress

In addition to *cis*- and *trans*-regulation, lncRNAs can also execute their functions as miRNA targets [9]. As tens of plant miRNAs have been functionally characterized, their roles can be applied to speculate the functions of lncRNAs. In this work, a total of 162 lncRNAs were identified as potential targets of 388 miRNAs. Specifically, one lncRNA, TCONS_00033722, was commonly targeted by six miRNAs including miR156, miR169, miR171, miR393, miR397, and miR398 (Fig. 8a), which were functionally conserved and responsive to salt stress [22]. The coordinated expression patterns of TCONS_00033722 and these miRNAs were further investigated by qRT-PCR (Fig. 8b). Interestingly, five of these miRNAs (except miR397) have been demonstrated to be regulated by auxin signaling [35–37]. Moreover, a few genes referred to auxin signaling were also identified as targets of these miRNAs through miRNA target prediction (Fig. 8a and Table S4): e.g., Spipo13G0022900 encoded a homolog of auxin signaling F-box 2 (*AFB2*) and was targeted by miR393 as previously reported [35]; an auxin efflux carrier (*PIN1*) and an auxin-inducible transcription factor (*IAA4*) were both targeted by miR398; two auxin transporters (*PGP1* and *PGP111*) were targeted by miR397 and miR169, respectively, and *PGP1* can mediate cellular efflux of IAA and interact with PIN genes [38]. Together, these findings strongly suggest that TCONS_00033722 is a key lncRNA participating in duckweed salt stress response via miRNA-mediated auxin signaling.

Although no auxin-related genes were identified as targets of miR171 and miR156, these two miRNAs targeted several genes referred to cell wall (e.g., *SVL1* for miR171, and *FRA8*, *WAK5*, and *IRX3* for miR156), indicating that TCONS_00033722 also played an important role in cell wall related metabolism, in agreement with the roles of auxin in cell wall expansion [39].

MiR156 is known to participate in responses to various abiotic stresses including salt, drought, and cold [22]. In addition to TCONS_00033722, miR156 also targeted three lncRNAs (including TCONS_00018793, TCONS_00045706, and TCONS_00057092) in this work, together with two transcription factors (*MYB96* and *WRKY33*) involved in various abiotic stresses, highly indicating that these three lncRNAs were involved in miR156-mediated salt stress in duckweed. MiR393, a function-known miRNA in auxin signaling, also targeted TCONS_00045512, suggesting that this lncRNA might have a similar function in duckweed with the participation of miR393 (Fig. 8a).

Taken together, these results revealed a complex network of lncRNAs, miRNAs, and mRNAs in duckweed under salt stress, based on which a schematic model was proposed (Fig. 8c). However, the functions of lncRNAs were not fully demonstrated and need to be further studied.

## Conclusions

In summary, thousands of novel lncRNAs were systematically discovered in duckweed *S. polyrhiza* in response to salt treatment, and their basic features as well as potential functions were extensively investigated. To the best of our knowledge, this work is the first report on salt-responsive lncRNAs in duckweed, and the findings will provide useful foundations for in-depth functional characterization of duckweed lncRNAs upon salt stress. Specifically, the three lncRNAs of TCONS_00033722, TCONS_00044328, and TCONS_00059333 are crucial candidates participating in salt stress probably via lncRNA-miRNA interaction, thus their roles deserve to be further studied.

## Methods

### Plant materials and salt treatments

*S. polyrhiza* (strain no. 7498), whose genome sequence is available in the public database, was obtained from Dr. Eric Lam’s lab and used in this study. To reveal the influence of salt stress on duckweed growth, 0.5 g three-frond colonies were inoculated in 250-mL flasks containing 100 mL MH medium supplemented with 0, 50, 100, and 150 mM NaCl, respectively, and cultivated under 16 h daily photoperiod at 25 ± 1 °C with 40 μmol · m^− 2^ · s^− 1^ photosynthetic active radiation (PAR) as previously reported [40]. The experiments were performed with three replicates at the Biological Collection Centre of the Institute of Tropical Bioscience and Biotechnology, Hainan, China. Duckweed samples were collected at a total of eight time-points including 0, 3, 6, 12, 24, 48, 72, and 96 h after salt treatment, respectively, and relative growth rate was calculated as the ratio of fresh weight under salt treatment to that under normal condition (0 mM NaCl).

To explore the lncRNAs in response to salt stress, *S. polyrhiza* samples were respectively collected at 0, 6, 12, and 24 h after 100 mM NaCl treatment, and immediately frozen in liquid nitrogen and kept at − 80 °C until use.

### RNA extraction, ssRNA-seq library preparation and Illumina sequencing

Total RNA extraction and libraries preparation were performed as previously described [24]. Briefly, total RNA was isolated for each sample by using the RNAprep Pure Plant Kit (TIANGEN Biotech, China). RNA quality and integrity were evaluated using an Agilent Bioanalyzer 2100 (Agilent, USA) as well as a Nanodrop-2000 spectrophotometer (Thermo Scientific Inc., USA). The ssRNA-seq libraries were generated using Illumina TruSeq™ RNA sample prep Kit (Illumina, CA, USA) together with Ribo-Zero Magnetic kit for rRNA removal following the manufacturer’s recommendation, and then sequenced with Illumina Hiseq-4000 instrument using paired-end mode with reads of 150 bp in length. Each sample was repeated three times.

### LncRNA identification

Low-quality and contaminated reads were discarded from raw-data files to generate clean reads, which were subsequently aligned to the duckweed reference genome with Tophat 2.0 software [41] setting the options of ‘-library-type fr-firststrand’. Cufflinks software [42] was used to assemble reads into transcripts, and those transcripts identified in no less than two samples were kept for further analysis. Then, Fragments Per Kilobase per Million mapped reads (FPKM) was calculated to estimate the expression level.

As previously described [24], a vigorous pipeline was used for lncRNA identification: 1) the transcripts with ORF length > 300, transcript length < 200 bp, minimal reads coverage < 3, or that overlapped with protein-encoding genes on the same nucleotide strand were discarded; 2) the transcripts with protein-coding potential were also excluded according to the evaluation of Coding-Potential Assessment Tool (CPAT) [43], Coding Potential Calculator (CPC) [44], and Coding-Non-Coding Index (CNCI) [45]; 3) the transcripts with well-known protein domains were also removed based on the Pfam-hidden Markov models. The remaining transcripts were regarded as reliably expressed lncRNAs. Differentially expressed (DE) lncRNAs were determined by setting |log_2_fold-change| > 1 and false discovery rate (FDR) < 0.05.

### LncRNA target prediction and enrichment analysis

To identify the targets of lncRNAs in trans-regulation, the expression profiles of DE lncRNAs and DE genes were pooled together to form a matrix for co-expression analysis according to the manual of WGCNA [46]. The lncRNAs and genes having similar expression trends were assigned into the same group (module) and potentially in trans-acting regulation. To reveal the potential function of lncRNAs in trans-acting regulation, duckweed genes were annotated and grouped into functional categories according to the MapMan classification system [47]. The significantly enriched functional categories were determined by the Fisher’s exact test as described previously [48].

To identify the targets of lncRNAs in cis-regulation, DE lncRNAs and their neighboring protein-encoding genes, which were placed 10 k/100 k up- and down-stream of these lncRNAs, were chosen to conduct co-expression analysis. The closely located and highly co-expressed lncRNA-mRNA pairs were regarded in cis-acting regulation.

### LncRNAs functioning as miRNA target

The lncRNAs acting as miRNA targets were analyzed by uploading the DE lncRNAs identified in this study and the miRNAs derived from miRBase v22 to psRNATarget [49]. The pairing regions of lncRNA-miRNA were allowed < 3 mismatches and G/U pairs according to the principles described by Wu et al. [10]. The interaction networks of lncRNAs, miRNAs and genes were imported into Cytoscape [50] for visualization.

### Differential expression analysis of genes

The bam files generated by Tophat 2.0 [41] were used as the input for Cufflinks v2.1.1 [42] to obtain the raw count data, which were subsequently subjected to library-size normalization by edgeR software [51]. DE genes were determined by DESeq [52] setting |log_2_fold-change| > 1 and FDR < 0.05.

### qRT-PCR analysis

The RNAprep Pure Plant Kit (TIANGEN Biotech, China) was applied to isolate total RNA for each sample. The PrimeScript RT reagent Kit containing gDNA Eraser (TaKaRa, China) was utilized to perform reverse-transcription of the first-strand cDNA. To validate the ssRNA-seq results, in total ten DE lncRNAs and five co-expressed genes were chosen to conduct qRT-PCR analysis using SYBR Premix Ex Taq (TaKaRa, China). In addition, the small RNAs were also reverse-transcribed into cDNAs using miRcute Plus miRNA First-Strand cDNA Kit (TIANGEN Biotech, China), and the expression of three miRNAs was validated by qRT-PCR using miRcute Plus miRNA qPCR Kit (SYBR Green, TIANGEN Biotech, China). The primers were listed in Table S1. qRT-PCR was conducted on a Stratagene Mx3000P instrument (Stratagene, USA) with the following procedures: 20 s at 95 °C; then 38 cycles of 10 s at 95 °C and 30 s at 60 °C. The amplification specificity was verified by a thermal denaturing step to generate melt curves. The 18S rRNA and U6 genes were used as the endogenous control. The relative expression level was calculated using the 2^-ΔΔCt^ method, and each sample was repeated at least three times.

## Supplementary information

**Additional file 1: Figure S1** The bioinformatic pipeline used for lncRNA identification in this work.

**Additional file 2: Table. S1** qRT-PCR primers and the correlation between qRT-PCR and ssRNA-seq. **Table. S2** Summary of 42 lncRNA-mRNA pairs in cis-acting regulatory relationship. **Table. S3** Summary of lncRNAs acting as miRNA targets. **Table. S4** Summary of genes presented in the lncRNA-miRNA-mRNA network of Fig. 8a.

## Data Availability

The generated ssRNA-seq data were deposited in NCBI-SRA database under the accession of PRJNA563960.

## Abbreviations

ABA: Abscisic acid
APX: Ascorbate peroxidase
CAT: Catalase
CNCI: Coding-Non-Coding Index
CPAT: Coding-Potential Assessment Tool
CPC: Coding Potential Calculator
DE: Differentially expressed
FDR: False discovery rate
FPKM: Fragments Per Kilobase per Million mapped reads
H_2_O_2_: Hydrogen peroxide
lincRNAs: Long intergenic noncoding RNAs
lncNATs: Long noncoding natural antisense transcripts
lncRNAs: Long noncoding RNAs
MDA: Malondialdehyde
nt: Nucleotides
PAR: Photosynthetic active radiation
Pi: Phosphate
POD: Peroxidase
PSI: Photosystems I
RGR: Relative growth rate
ROS: Reactive oxygen species
SOD: Superoxide dismutase
ssRNA-seq: Strand-specific RNA sequencing
