# Supplementary figures and images for: Genome-wide discovery and functional prediction of salt-responsive lncRNAs in duckweed
Source: BMC Genomics. 2020 Mar 5;21:212. doi: 10.1186/s12864-020-6633-x (PMC7059339; doi:10.1186/s12864-020-6633-x)

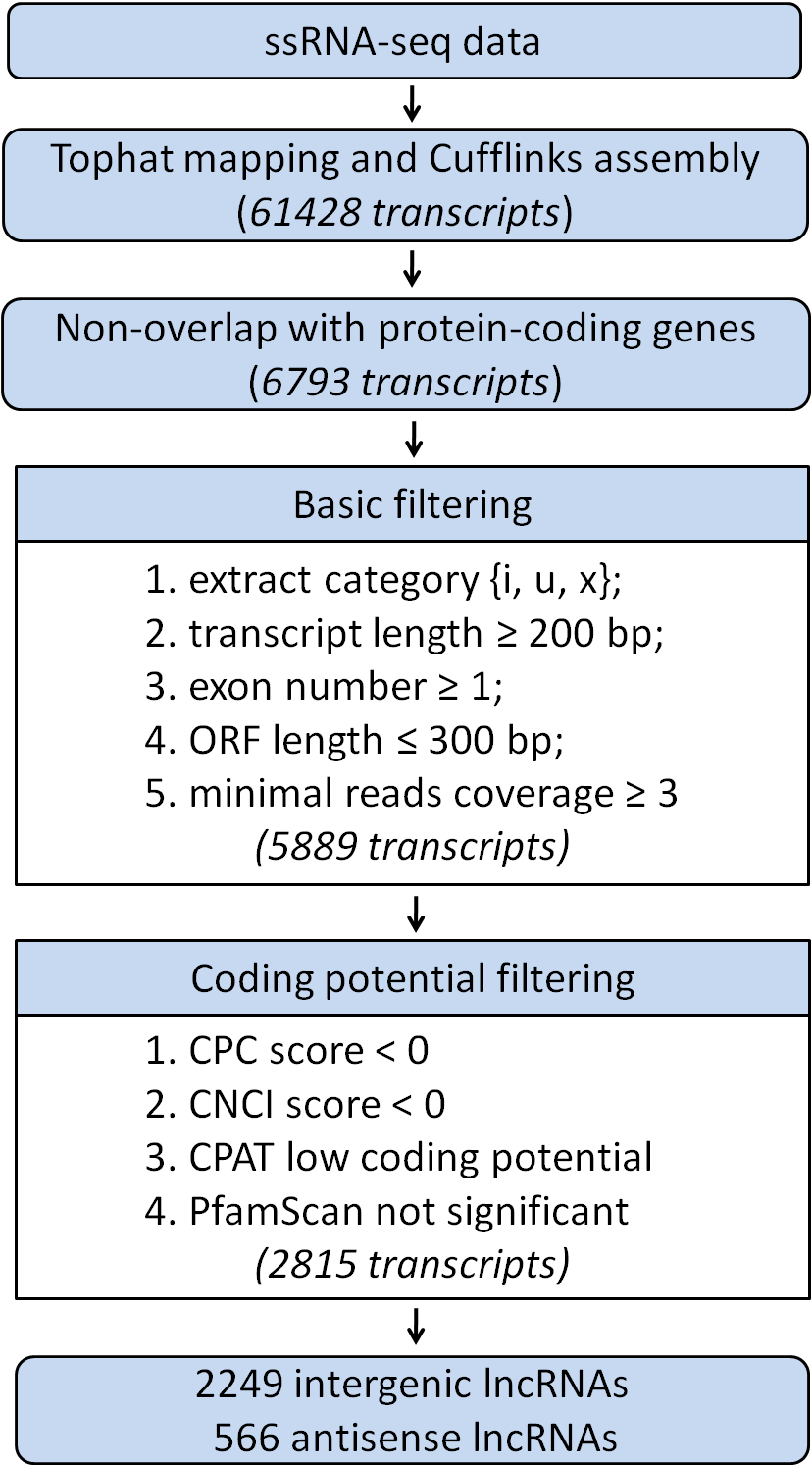

Supplement: Supplementary file 1 — Additional file 1: Figure S1 The bioinformatic pipeline used for lncRNA identification in this work. [file 12864_2020_6633_MOESM1_ESM.tif]
